# Research Progress on Neurodevelopmental Toxicity in Offspring after Indirect Exposure to PFASs in Early Life

**DOI:** 10.3390/toxics11070571

**Published:** 2023-06-30

**Authors:** Huai-Yu Zhuchen, Jie-Yu Wang, Xiao-Shan Liu, Yan-Wei Shi

**Affiliations:** 1 Faculty of Forensic Medicine, Zhongshan School of Medicine, Sun Yat-sen University, Guangzhou 510080, China; zhuchhy@mail2.sysu.edu.cn (H.-Y.Z.); wangjy79@mail2.sysu.edu.cn (J.-Y.W.); 2 Dongguan Key Laboratory of Environmental Medicine, The First Dongguan Affiliated Hospital, Guangdong Medical University, Dongguan 523808, China; 3 Guangdong Province Translational Forensic Medicine Engineering Technology Research Center, Guangzhou 510000, China; 4 Guangdong Province Key Laboratory of Brain Function and Disease, Zhongshan School of Medicine, Sun Yat-sen University, Guangzhou 510080, China

**Keywords:** PFAS, early life exposure, placenta, developmental neurotoxicity

## Abstract

Per- and polyfluoroalkyl substances (PFASs) are widespread environmental pollutants. There is increasing evidence that PFASs have various adverse health effects, including renal toxicity, metabolic dysfunction, endocrine disruption, and developmental toxicity. PFASs have been found to accumulate in the placenta, and some PFASs can cross the placental barrier and subsequently accumulate in the fetus via the maternal–fetal circulation. An increasing number of studies have shown that early life exposure to PFASs can affect fetal neurodevelopment. This paper reviews the characteristics of indirect exposure to PFASs in early life, the effects on neurodevelopment in offspring, and the possible mechanisms of toxic effects.

## 1. Introduction

Per- and polyfluoroalkyl substances (PFASs, C_n_F_2n+1_-R) refer to a range of chemicals that have been produced since the late 1940s. Due to the strong electronegativity and the small atomic size of fluorine, the perfluoroalkyl fraction (C_n_F_2n+1_-) has improved molecular properties compared to its hydrocarbon counterpart (e.g., stronger acidity, higher surface activity at very low concentrations, stability, and/or water and oil resistance). As a result, PFASs have been widely used in household products such as cosmetics, firefighting foams, and food packaging materials, as well as in printing inks, medical devices, oil production, mining, pesticide formulations, and industrial sectors such as the textile, leather, and clothing industries [1].

PFASs contain one or more carbon atoms, with a fluorine atom replacing the hydrogen atom. The compounds contain a carbon atom that is part of nF_2N+1_-R, where R represents other functional groups (e.g., sulfonate, carboxylic acid). The perfluorinated or polyfluoroalkyl moiety is linked with extremely strong carbon–fluorine bonds, making PFASs highly chemically and thermally stable, lipophilic, and hydrophilic. These physicochemical properties make PFASs ideal for use in surfactants and surface protection products [2]. PFAS can be grouped into two broad categories: non-polymeric and polymeric molecules. Non-polymeric PFAS can be further subdivided into two groups represented by perfluoroalkyl and polyfluoroalkyl substances. The former includes molecules wherein the hydrophobic carbon chain is totally fluorinated with the exception of the terminal end, which hosts a polar functional group such as carboxylate (COO^−^), sulfonate (SO^3−^) or phosphate (OPO^3−^) which confers hydrophilicity [1]. PFASs and their breakdown products, perfluoroalkyl acids (PFAAs), are highly persistent and difficult to degrade in the environment, including in groundwater, resulting in ongoing exposure of the global population and some local/regional communities. Common PFAAs include long-chain PFAAs such as perfluorooctane sulfonic acid and its derivatives (PFOS), perfluorooctanoic acid (PFOA), perfluoroundecanoic acid (PFUnA), perfluorononan-1-oic acid (PFNA), and perfluorodecanoicacid (PFDA), and short-chain PFAAs such as perfluorohexanoic acid (PFHxA), perfluorobutanoic acid (PFBA), perfluorobutane sulfonic acid (PFBS), perfluorohexane sulfonic acid (PFHxS) and perfluoroheptanoate (PFHpA) [3]. More information about PFAAs can be found in Table 1. In addition, hexafluoropropylene oxide dimer acid (HFPO-DA, also known as GenX) is a new alternative to PFOA that is on the market. Traditional PFASs, mostly long-chain PFAAs, have been phased out and replaced with GenX and other short-chain PFAAs for reasons such as excessive toxicity [4,5].

PFASs have been identified as persistent organic pollutants [6]. New or traditional PFASs have been detected in all types of environmental media, including soil, water, air, and biota [3,7,8]. Near contaminated sites, drinking water accounts for 75% of total PFASs exposure [4]. For atmospheric contamination, PFASs can be transferred from the sea surface to sea spray aerosol particles by bubble bursting, and PFASs emitted to the atmosphere in the gas phase can be separated and adsorbed to particulate matter by gas particles [9]. PFASs are widely used in common commercial and household products, and humans can be exposed daily through oral, respiratory, or dermal contact [10]. In China, PFOA, PFOS, PFNA, PFBS, PFHpA, and PFHxS were detected predominantly in drinking water and indoor dust samples, with higher concentrations of short-chain PFASs than long-chain PFASs. Higher concentrations of PFASs were observed in tap and filtered water than in bottled water [3]. Groundwater from PFAS-contaminated water sources has been inadvertently used as a drinking water supply in Sweden, Germany, the UK, and Italy [11,12,13,14].

Studies have shown that PFASs are a class of multisystem toxicants that have effects on the liver, kidneys, immune system, thyroid, and cardiovascular and reproductive systems [15,16,17]. Some PFASs are structurally similar to natural fatty acids and tend to accumulate in the serum, lungs, kidneys, liver, and brain. Animal studies have shown that PFOS accumulates in the lungs, kidneys, spleen, heart, and brain in mice, causes damage to the liver and border region of the heart, and affects hepatic glycerophospholipid metabolism and sphingolipid metabolism [18]. In addition, after 28 days of exposure to PFOA, abnormal liver function and kidney function, together with a decrease in the activity of enzymatic antioxidants (CAT, GPx, and SOD) in liver and kidney tissues, were observed in rats [19]. Global epidemiological studies have reported the adverse human health effects of PFASs from environmental contamination, including infertility, steroid hormone disruption, thyroid, liver, and kidney disease, metabolic dysfunction, and increased cancer risk [2,7,20,21]. Based on the prevalence of PFAS exposure in the population and the adverse effects, this review focused on summarizing the neurodevelopmental effects and possible mechanisms of indirect exposure to PFASs in early life.

## 2. Characteristics of PFAS Exposure in Early Life

Studies have shown that PFASs can accumulate in the placenta. PFASs were detected in 120 human placental tissue samples collected in the USA between 2010 and 2011, and the highest levels of PFOS, PFOA, PFNA, and PFDA were found in the placenta, with median concentrations of 0.95, 0.27, 0.11, and 0.06 (ng/g), respectively. There was no significant difference between the placental concentrations on the maternal side and those on the fetal side [22]. In the vicinity of the Fuxin Fluorine Chemical Industrial Park in China, 50 paired maternal serum, placental tissue, and neonatal serum samples were selected; PFAS—including PFBS, PFOA, and PFBA—were shown to be the major contaminants, with median placental tissue concentrations of 33, 21, and 26 ng/g, respectively [23]. Mamse et al. investigated the concentrations of PFOS, PFDA, PFNA, PFDA, PFUnA, and PFHxS in placental, maternal serum, and fetal serum samples. They found that these PFASs were detected in all samples and that the concentrations of PFASs in the placental samples were similar to those in the fetal serum samples, all of which were lower than the concentrations in the maternal plasma samples. During pregnancy, the ratio of PFOS, PFOA, and PFNA concentrations in the placental and serum samples increased, suggesting that the accumulation of these substances in the placenta increased [24].

PFASs can cross the placenta from the mother to the fetus and accumulate in the fetus. In a study of PFAS exposure in early pregnancy among women in the Boston area Project Viva cohort, higher levels of PFOS, PFOA, PFHxS, and PFNA were found in nulliparous women than in multiparous women, possibly due to the transfer of PFASs across the placenta from the women to their fetuses during pregnancy [25]. Another study found that certain PFAS levels were lower in the blood plasma and/or cord plasma of mothers with three or more children than in women of the same age with fewer children. A comparison of maternal blood and cord blood samples showed that mothers could transfer significant amounts (40% or more) of PFASs across the placenta to their babies and that this transfer occurs in every pregnancy [26,27]. In addition, one study found higher levels of PFASs in the placentas of preterm babies than in the placentas of full-term babies [28]. In term infants, placental PFAS concentrations were similar to fetal organ concentrations, and the proportions of PFOS, PFOA, and PFNA in the placenta and maternal serum samples increased during the third trimester. Toxic accumulation may gradually increase due to increased plasma volume, placental bioaccumulation, or blood dilution [29,30].

Regarding the specific efficiency of the transfer of PFASs across the placenta from the mother to the fetus, studies have shown that PFASs mainly bind to human serum proteins and liver fatty acid transport proteins in the plasma to form complexes, and these complexes cannot cross the placental barrier to the fetus. Thus, it is mainly the free PFASs in the maternal circulation that enter the fetus via the umbilical cord blood. The placental transfer efficiency of PFASs is related to the binding capacity of PFASs and proteins or the dissociation constant of the complex, and the length of the PTE and PFAS chains is an inverted U-shaped curve [31]. Ke Gao et al. analyzed the concentrations of 21 PFASs in 132 pairs of maternal and cord serum samples collected from residents of Beijing, China, and calculated the placental transfer efficiency (PTE, PFAS concentration in cord blood plasma/PFAS concentration in maternal plasma) for each PFAS PFOS (44%), PFOA (83.2%), PFBA (146%), PFBS (97%), PFDA (44%), and PFHxA (110%) [32]. Another cohort study also showed that novel PFASs may be more likely to cross the placenta than conventional PFASs [33].

In addition to the placenta, breast milk is an important route for the excretion of PFASs in postpartum women [34,35,36]. Studies have shown that women who have never breastfed have higher blood levels of PFASs, especially PFOS and PFOA, than women who have breastfed. Among children born in known PFAS-contaminated areas, breastfed children have elevated serum concentrations of PFASs other than PFHxS compared to formula-fed children [37]. For most PFASs, their concentration levels in vivo follow the order of maternal serum > cord serum > breast milk. Breast milk transfer efficiencies were 1–2 orders of magnitude lower than transplacental transfer efficiencies for most PFASs, except for PFBS, which showed high transfer efficiencies across both the placenta and breast milk [38]. Using models to estimate the accumulation of toxicants in a fetus after maternal exposure to three PFASs (PFOA, PFOS, and PFHxS), a comparison of the two pathways of placental transfer during pregnancy and postpartum transfer through breastfeeding showed that breastfeeding led to significantly increased PFAS concentrations in infants, who may have even higher PFAS concentrations than their mothers [27]. Although the mechanism of PFAS transfer through breast milk is unclear, the structure of PFASs is similar to that of the long-chain fatty acids found in breast milk. PFASs may also be transferred through breast milk after binding to proteins in breast milk [39,40].

## 3. PFAS Exposure in Early Life Induced Neurodevelopmental Toxicity in Offspring

Preliminary population studies suggest two potential mechanisms for PFASs entry into the brain: disruption of tight junctions or reliance on blood-brain barrier (BBB) transporter proteins. Both long- and short-chain PFASs are capable of competitively binding proteins for uptake into tissues by circulating transport and intracellular delivery [41,42]. Similarly, in analyses that simulate prenatal maternal PFAS serum concentrations as intrauterine exposure, an association between prenatal PFHxS and PFOS exposure and increased odds of a child being diagnosed with autism spectrum disorder was observed [43]. Studies have also examined the association between early exposure to PFOS and PFOA and attention deficit hyperactivity disorder (ADHD), including nine European population studies involving 4826 mother–child pairs, which found a total of 399 children classified as having ADHD that was not associated with early exposure to PFOS or PFOA. However, potential differential effects of PFAS exposure on child sex and maternal education levels were found in hierarchical models [44,45]. Studies of ADHD have sometimes yielded different results in different populations. In the Hokkaido study, higher maternal serum levels of PFASs during pregnancy were found to be associated with a lower risk of ADHD symptoms in children by the age of 8 years [46]. The Odense Child Cohort Study found no association between prenatal PFAS exposure and autism in children [47].

There is evidence that the neurological effects of PFAS exposure during development are more pronounced than those of exposure in adulthood. If mothers are exposed to PFOS during pregnancy, offspring development can be affected, particularly motor development at the age of 2 years [48]. In China, using data from the Laizhou Wan Birth Cohort, prenatal exposure to PFBS or PFHxS was found to be negatively associated with neurodevelopmental scores at 1 year of age, particularly in the gross motor domain. Another study noted that poor gross motor skills in the first year of life had been identified as a predictor of ADHD [49,50]. Similar cohort studies have also found that exposure to PFASs (including PFOA, PFOS, and PFHxS) during gestation and childhood may be associated with reduced visual-motor skills in children [51]. Because PFAS exposure is ubiquitous in the lives of pregnant women and children, even small adverse effects of PFASs on cognitive development can result in a substantial neurodevelopmental burden on a large and cumulative scale over time.

In animal studies, PFASs have also shown significant neurodevelopmental toxic effects, mainly in cognitive function, learning, and memory, as well as motor function, which are often achieved by altering neurotransmitters. During the vulnerable period of postnatal brain development, a single exposure to PFHxS caused irreversible neurotoxicity in cognitive function in mice, manifested by changes in spontaneous adult behavior, and this reduced cognitive function may be associated with changes in the function of the cholinergic system [52]. In mice given a high oral dose of PFOS (11.3 mg/kg) for 24 h, a decrease in the cortical expression of acetylcholinesterase and nicotinic acetylcholine receptor genes was observed, along with complex changes in acetylcholine receptor gene transcription [53].

Pre- or postnatal exposure of mice to PFOS resulted in reduced spatial learning and memory in offspring, particularly those exposed prenatally [54]. Similarly, altered levels of CaMKII, GAP-43, synaptophysin, and tau protein were found in mice exposed orally to 9.2 mg/kg of PFHxS, whereas animals exposed to 6.1 mg/kg of PFHxS only showed changes in CaMKII and tau protein, which are proteins and factors closely related to neuronal function [55]. Learning and memory deficits also occur in mice that are postnatally exposed to PFOS. Neurotransmitter levels of Glu and GABA were found to be significantly elevated in the dorsal hippocampus by in vivo microdialysis, but mRNA levels of GABA and glutamate receptor genes did not change much at the transcriptional level [56].

Mice that were prenatally exposed to PFOS showed a clear trend toward hyperactivity after birth, as well as dose-dependent changes in anxiety. Monitoring changes in metabolites in the mouse brain revealed increased levels of GABA, taurine, Gly, Met, Pro, Ser, and T4-hydroxyproline [57]. After ingesting PFOS through breast milk 1–14 days after birth, neonatal mice underwent the Object Location Test (OLT), Object Recognition Test (ORT), and Paired Visual Discrimination (VD) at 8–10 weeks of age, and PFOS was found to impair memory for object location and recognition, as well as the visual-discrimination learning ability [56]. Whole-cell membrane clamp recordings of Purkinje cells showed that short-term plasticity and long-term plasticity in parallel fiber Purkinje cells were disrupted by PFOS exposure. Western blot analysis showed that PFOS exposure resulted in increased expression levels of synthetic protein-binding protein 1 (Munc18-1) and glutamate metabolizing receptor 1 (mGluR1), which subsequently affected synaptic plasticity and motor coordination in neurons [58]. Failure to inflate the swim bladder, abnormal caudal-ventral flexion, and hyperactivity at nonteratogenic concentrations were found in adult zebrafish following early developmental exposure to PFHxS or PFOS [59].

Animal studies have confirmed that abnormal behavioral changes in mice exposed to PFAS in early life include changes in motor development, memory capacity, and cognitive levels. The changes are mainly due to altered expression of neurotransmitter receptors, synaptic plasticity, and related proteins in nerve cells, which subsequently affect brain function, spatial learning, and memory capacity. The levels of amino acids and neurotransmitters in the brain and their subsequent effects on metabolic pathways are altered by PFAS exposure in early life, and such changes occur in different brain regions, culminating in the corresponding changes observed in the behavior of the mice. Thus, it is clear that the mechanisms of neurodevelopmental toxicity following early-life PFAS exposure deserve in-depth investigation.

## 4. Mechanisms of Neurodevelopmental Toxicity after Exposure to PFAS in Early Life

### 4.1. PFASs Cause Neurodevelopmental Toxicity through Effects on Placental Thyroid Hormones

The effects of PFASs on thyroid hormones (THs) have long been of interest. Thyroxine plays a critical role in neurodevelopment by regulating dendritic processes, axonal growth, synaptogenesis, neuronal migration, and myelin formation. In early pregnancy, fetuses are known to be largely dependent on maternal THs, and the disruption of maternal TH homeostasis affects both maternal and fetal health. This also affects normal fetal neurodevelopment through restricted dendritic and axonal growth, abnormal nerve location, and altered synaptic function [60]. In a prospective cohort study in a Japanese region, maternal serum PFOS and PFOA concentrations in the first trimester, third trimester, and one week after delivery, maternal and infant thyrotropin (TSH), and free thyroxine (FT_4_) levels showed that maternal TSH levels decreased, but infant TSH levels increased after PFOS exposure in the first trimester of pregnancy [61]. When samples were taken from mothers and fetuses in early pregnancy, PFOS concentrations in the mothers were positively correlated with total T_4_ and PFOA concentrations and negatively correlated with FT_4_ levels. PFHxS concentrations in infants were negatively correlated with T_4_ levels [62]. In the Canadian Birth Cohort Study, there was a consistent negative correlation between maternal PFHxS concentrations and FT_4_ concentrations at all time points between pregnancy and 4 months postpartum and a positive correlation between maternal PFHxS concentrations and TSH concentrations in early pregnancy, which is also a critical period for fetal neurodevelopment [63]. Another meta-analysis found an association between exposure to PFOS, PFOA, and PFDA during pregnancy and maternal TSH levels, with pregnant women’s TH levels being more sensitive to the effects of PFAS exposure during the first 6 months of pregnancy [64]. In studies of Chinese populations, prenatal exposure to PFASs (especially PFBS and PFHxS) was negatively associated with TH levels (especially TSH and FT_4_), and TH levels (especially TSH and FT_4_) were positively associated with neurodevelopment (especially gross motor and social development). The associations were more pronounced in male infants after sex stratification, and the underlying mechanism for this sex difference is unclear. The placental permeability of PFBS and PFHxS is high, and cord serum TSH and FT_4_ may be involved in the association of PFBS with gross motor function and adaptation [49]. These studies suggest a negative association between maternal PFAS concentrations and maternal FT_4_ and TSH levels in early pregnancy and a positive association between maternal PFAS concentrations and infant TSH levels at environmental levels of PFAS exposure, which is generally consistent with conclusions from animal models and in vitro experiments.

Animal studies have confirmed that decreases in serum total T_4_ and 3,3′,5-triiodothyronine (T_3_) levels, together with slight increases in TSH and thyrotropin-releasing hormone levels, were observed in fetal, adolescent, and adult mice born to mothers exposed to PFBS during pregnancy [55]. Similarly, T_4_ and T_3_ levels were reduced in mice exposed to PFOS prenatally or with a single exposure to PFOS postnatally, and this change was time-dependent [56]. In zebrafish, transthyretin (TTR) transcription was significantly downregulated in a concentration-dependent manner by single exposures to 5.2 and 5.6 mg/L of PFOS during early embryonic development [57]. The above studies show that the production, transport, release, and metabolism of thyroid hormones are affected in both mothers and offspring exposed to PFASs, with the offspring exhibiting features of hypothyroxinemia and associated neurodevelopmental defects in early life.

In vitro cell experiments have been used to investigate the possible mechanisms by which PFASs interfere with thyroid hormone function through the placenta. It was found that the concentrations of fetal THs change during pregnancy. This change is not synchronized with the concentrations of THs in maternal serum, suggesting the presence of a maternal–fetal mechanism for the homeostatic regulation of thyroid hormones in the placenta. The circulating pathway of THs from the mother to the fetus is regulated by plasma membrane transporters, enzymes, and carrier proteins [65]. This suggests that PFASs may affect the mother–fetus cycle by affecting these enzymes and transporters, which are highly expressed in the placenta, leading to changes in TH levels in offspring. PFOS has been found to prevent sodium iodide from the uptake of transporter-mediated iodide, thereby reducing intracellular iodide concentrations in iodine-containing cells and affecting TH synthesis [66]. In vitro experiments have also shown that PFDA can compete for T_4_ binding to TTR, temporarily increasing circulating levels of FT_4_, and then regulating T_4_ synthesis through negative feedback on the HPT axis, which results in increased TSH levels and decreased free thyroxine levels in the fetus [67,68].

### 4.2. PFAS Causes Neurodevelopmental Toxicity by Affecting Placental Neurotrophic Factor Secretion

The direct toxic effects of PFASs on neurons have been well studied, but there are relatively few studies focusing on changes in the neurons of offspring following maternal PFAS exposure. A study of the human placenta showed that brain-derived neurotrophic factor (BDNF) receptors are present in different regions of human placental villi, suggesting that BDNF signaling may have distinct functions in placental development. Observations of early and full-term human placentas and trophoblast cells showed that the BDNF signaling pathway is affected by PFNA exposure but not by exposure to a mixture of PFOS, PFOA, PFBS, and PFAS [69]. In 725 pregnant women from the Shanghai birth cohort, prenatal exposure to PFHxS was associated with elevated levels of BDNF in umbilical cord blood, particularly in male fetuses [70]. This suggests that PFAS exposure may cause neurodevelopmental toxicity via neurotrophic factors in the placenta.

By examining changes in proteins and neurotrophic factors associated with neuronal development in animal studies, it was found that pups exposed to PFOS after birth had reduced habituation to their new home environment, altered spontaneous behavior, and decreased levels of GAP-43, NCAM1, nerve growth factor (NGF), and BDNF proteins in the hippocampus at Day 35 after birth. Levels of these key proteins associated with synaptic plasticity were inhibited by PFOS exposure, and some changes in the corresponding gene levels occurred. The gene levels of *gap-43*, *ncam1*, and *bdnf* increased during early offspring development, but this increase was not sustained as PFOS exposure increased, nor were the levels of the corresponding proteins. This suggests that PFOS may also affect the subsequent transcription and translation of genes, leading to protein deletion [54]. Another study showed that GAP-43 mRNA levels were significantly higher on Day 14 of life in pups that were prenatally exposed to high doses of PFOS. The expression of BDNF, GAP-43, and NCAM1 was significantly higher in the hippocampus than in the cortex, a change that could also be caused by a decrease in thyroxine levels following PFOS exposure [71]. GAP-43 and NCAM1 are membrane proteins that are essential for neuronal development, and NGF and BDNF are important members of a family of neurotrophic factors that regulate neuronal survival, differentiation, growth, and hippocampal long-term potential.

The changes in neurotrophic factors following PFAS exposure observed in the above studies may ultimately lead to changes in the synaptic plasticity of neurons, resulting in behavioral abnormalities.

## 5. PFAS Causes Neurotoxicity by Altering Nerve Cell Function

Neurotransmitters have been studied more intensively in in vitro experiments following PFAS exposure. Molecular mechanism studies have shown that PFASs can affect neuronal differentiation. When rat stem cells were differentiated for five days after two days of exposure to PFOS, the cells were found to be biased toward differentiation into neurons or oligodendrocytes and less so toward differentiation into astrocytes, suggesting that PFOS may alter stem cell differentiation and that this differentiation trend may have significant effects on overall brain development [72]. In astrocytes, PFOS altered extracellular Glu and Gln concentrations, reduced glutamine synthetase activity, and impaired the gene expression of glutamine synthetase, glutamate, and glutamine transporter proteins [73]. It has been suggested that PFOS exposure may alter the transport mechanism of glutamate release via glutamine recycling (glutamate–glutamine cycle) in astrocytes. This cycle prevents cellular excitotoxicity by maintaining extracellular Glu levels within a homeostatic range, and when the cycle is disrupted, Glu increases, and Gln decreases.

PFASs can induce cellular oxidative stress and damage cells in several ways [7], and the exposure of primary rat hippocampal neurons and astrocytes to PFOS results in redox imbalance, increased apoptosis, and abnormal autophagy [73]. An in vitro study found that ROS were generated in neurons and astrocytes after 5 min of exposure to PFOA, with dopamine neurons also affected by ROS and mitochondrial damage [73,74]. There are also some differences in the redox outcomes caused by different types of PFASs, with a significant decrease in the total antioxidant capacity (TAC) in cells exposed to PFOA and a trend toward increased TAC in cells exposed to PFHxS and PFNA and a nonsignificant decrease in TAC in cells exposed to PFOS [75,76]. PFOS may also induce apoptosis in cerebellar granule cells via the ROS-mediated PKC signaling pathway [77]. Following PFAS stimulation, intracellular ROS increase, and redox imbalance and calcium ions also change. Downstream calcium targets associated with PFOS exposure include CaMKII, CREB, and GAP-43, which are proteins that are involved in important aspects of neurodevelopment. Excessive activation of calcium signaling can also lead to activation of the NMDA apoptotic pathway [78]. In mice, postnatal PFHxS exposure induces sustained GAP-43 and CaMKII downregulation via the NMDA receptor-mediated PKC (α and δ)-ERK/AMPK pathway, leading to neurodevelopmental toxicity [79].

PFASs may also affect neuronal development and function through epigenetic changes. PFASs alter DNA methylation levels in offspring, which in turn affects RNA and protein expression, and this effect can be progressively amplified by environmental and behavioral changes, acting at key points in neural development. For example, for BDNF, which is associated with neuronal cell growth, PFOS increased the expression of BDNF-associated miRNAs in human neuroblastoma cells and altered the methylation levels of BDNF promoters I and IV, significantly reducing BDNF mRNA expression and protein levels [80]. BDNF plays an important role in the survival and differentiation of the central nervous system, regulating learning and memory levels, and studies suggest that regulation of the methylation of the BDNF gene promoter and an increase in BDNF-related microRNAs may be the molecular basis for the mechanism of PFOS-induced neurotoxicity [81]. An epidemiological study showed an association between PFAS (PFHxS, PFOS, PFNA, and PFDA) concentrations in maternal blood in early pregnancy and total DNA methylation levels in newborns and a mediating effect of 5-methylcytosine (5-mC) and 5-hydroxymethylcytosine (5-hmC) levels in the relationship between PFNA and PFUnDA exposure and reduced gestational age [80].

**Figure 1:**
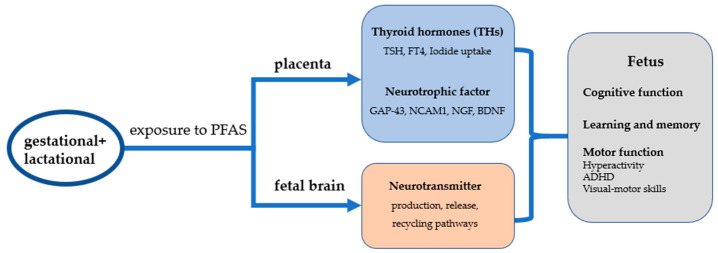
Mechanisms of neurodevelopmental toxicity after exposure to PFAS in early life.

Therefore, early life exposure to PFASs may affect cell function through oxidative stress and epigenetic aspects of neuronal cells, coupled with altered neurotransmitter production, release, and recycling pathways, leading to impaired neuronal and glial cell development, which results in neurodevelopmental toxicity in offspring exposed to PFASs in early life (see Figure 1).

## 6. Outlook

As science and technology advance, the use of PFASs will become more widespread. Research on the pathways of maternal effects on offspring neurotoxicity after PFASs exposure seems necessary at present, but there are many problems in this research.

Existing environmental epidemiological studies have reported inconsistent or contradictory findings due to sample size problems or confounding factors. Future studies could increase the sample size to fully account for confounding factors, such as the role of other environmental contaminants, and clarify the causal relationship between early PFASs exposure and neurodevelopmental abnormalities in offspring.

Existing animal toxicity tests tend to use high doses of contaminants that do not match actual population exposures, increasing the uncertainty in extrapolating from high to low doses. At the same time, single-substance toxicity tests are often used, which do not reflect the characteristics of actual combined multisubstance exposures. Low-dose toxicity experiments for PFASs and the combined exposure effect are the future directions of research.

There are few studies on the mechanisms of neurotoxicity in offspring with early-life exposure to PFASs. PFASs may affect the development of neurons in offspring by interfering with placental function, or PFASs may directly affect neurodevelopment, which needs to be further elucidated.

Current research on PFASs has generally focused on PFOS and PFOA, but the use of these two substances has gradually declined due to excessive toxicity; these substances have been replaced by newer PFASs, which may also receive more attention in future studies. The accumulation of PFASs and other types of toxicants in the body is also noteworthy due to the complexity of the compounds used in industrial production.

## Figures and Tables

**Table 1:** Basic properties of common PFAAs.

Type/Characteristic	Structural Formula	CAS	Molecular Weight (g/mol)	pKa	Solubility
perfluorobutanoic acid (PFBA)	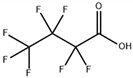	375-22-4	214.04	pK1:0.17 (25 °C)	Chloroform: soluble; Methanol: soluble
perfluorobutane sulfonic acid (PFBS)	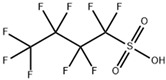	375-73-5	300.1	−3.57 ± 0.50 (Predicted)	Soluble in water
perfluorodecanoicacid (PFDA)	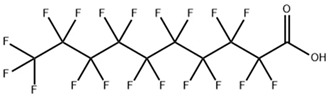	335-76-2	514.08	0.52 ± 0.10 (Predicted)	methanol: soluble 10%
Perfluoroheptanoate (PFHpA)	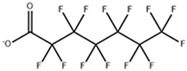	120885-29-2	363.05	-	-
perfluorohexanoic acid (PFHxA)	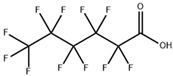	307-24-4	314.05	0.42 ± 0.10 (Predicted)	water: insoluble
perfluorohexane sulfonic acid (PFHxS)	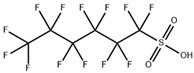	355-46-4	400.11	−3.34 ± 0.50 (Predicted)	DMSO (Slightly), Methanol (Slightly)
perfluorononan-1-oic acid (PFNA)	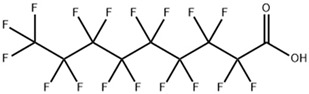	375-95-1	464.08	0.52 ± 0.10 (Predicted)	Acetone (Slightly), DMSO (Slightly), Methanol (Slightly)
perfluorooctanoic acid (PFOA)	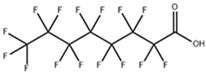	335-67-1	414.07	0.50 ± 0.10 (Predicted)	Water: 3.4 g/L
perfluorooctane sulfonic acid and its derivatives (PFOS)	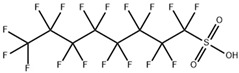	1763-23-1	500.13	−3.27 ± 0.50 (Predicted)	Ethanol: 10 mg/mL
perfluoroundecanoic acid (PFUnA)	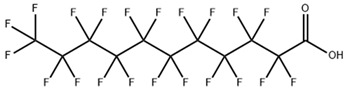	2058-94-8	564.09	0.52 ± 0.10 (Predicted)	DMSO (Slightly), Methanol (Slightly)

## Data Availability

Not applicable.

## Abbreviations

ADHD	attention deficit hyperactivity disorder
BBB	blood–brain barrier
BDNF	brain-derived neurotrophic factor
CaMKII	calcium/calmodulin dependent protein kinase II
CAT	catalase
CREB	cyclic-AMP response binding protein
FT4	free thyroxine
GABA	Gamma amino butyric acid
GAP-43	Growth associated protein-43
GenX	2,3,3,3-tetrafluoro-2-(heptafluoropropoxy) propanoate
GGT	γ-Glutamyl Transferase
Gln	Glutamine
Glu	Glutamic
GPx	Glutathione peroxidase
HFPO-DA	hexafluoropropylene oxide dimer acid
HPT	axis Hypothalamic-pituitary-thyroid axis
NCAM1	Neural Cell Adhesion Molecule 1
NGF	nerve growth factor
NMDA	N-methyl-D-aspartic acid
PFAAs	perfluoroalkyl acids
PFAS	per- and polyfluoroalkyl substances
PFBA	perfluorobutanoic acid
PFBS	perfluorobutane sulfonic acid
PFDA	perfluorodecanoicacid
PFHpA	perfluoroheptanoate
PFHxA	perfluorohexanoic acid
PFHxS	perfluorohexane sulfonic acid
PFNA	perfluorononan-1-oic acid
PFOA	perfluorooctanoic acid
PFOS	perfluorooctane sulfonic acid and its derivatives
PFUnA	Perfluoroundecanoic acid
PKC	protein kinase C
PTE	placental transfer efficiency
ROS	Reactive Oxygen Species
SOD	Super Oxide Dismutase
T3	3,3′,5-triiodothyronine
T4	thyroxine
TAC	total antioxidant capacity
TH	thyroid hormone
TSH	thyrotropin
TTR	transthyretin
